# Comprehensive Metabolomic Analysis in Blood, Urine, Fat, and Muscle in Men with Metabolic Syndrome: A Randomized, Placebo-Controlled Clinical Trial on the Effects of Resveratrol after Four Months’ Treatment

**DOI:** 10.3390/ijms18030554

**Published:** 2017-03-04

**Authors:** Anne Sofie Korsholm, Thomas Nordstrøm Kjær, Marie Juul Ornstrup, Steen Bønløkke Pedersen

**Affiliations:** Department of Endocrinology and Internal Medicine, Aarhus University Hospital and Institute of Clinical Medicine, Aarhus University, DK-8000 Aarhus C, Denmark; thomas.kjaer@clin.au.dk (T.N.K.); Marie.juul.ornstrup@clin.au.dk (M.J.O.); amtssp@gmail.com (S.B.P.)

**Keywords:** resveratrol, natural bioactive agents, metabolic syndrome, metabolomics, metabolite profiling, gut microbiome, obesity, insulin sensitivity, inflammation

## Abstract

Resveratrol possesses several beneficial metabolic effects in rodents, while the effects of resveratrol in humans remain unclear. Therefore, we performed a non-targeted comprehensive metabolomic analysis on blood, urine, adipose tissue, and skeletal muscle tissue in middle-aged men with metabolic syndrome randomized to either resveratrol or placebo treatment for four months. Changes in steroid hormones across all four matrices were the most pronounced changes observed. Resveratrol treatment reduced sulfated androgen precursors in blood, adipose tissue, and muscle tissue, and increased these metabolites in urine. Furthermore, markers of muscle turnover were increased and lipid metabolism was affected, with increased intracellular glycerol and accumulation of long-chain saturated, monounsaturated, and polyunsaturated (n3 and n6) free fatty acids in resveratrol-treated men. Finally, urinary derivatives of aromatic amino acids, which mainly reflect the composition of the gut microbiota, were altered upon resveratrol treatment. In conclusion, the non-targeted metabolomics approach applied to four different matrices provided evidence of subtle but robust effects on several metabolic pathways following resveratrol treatment for four months in men with metabolic syndrome—effects that, for the most part, would not have been detected by routine analyses. The affected pathways should be the focus of future clinical trials on resveratrol’s effects, and perhaps particularly the areas of steroid metabolism and the gut microbiome.

## 1. Introduction

Resveratrol is a stilbene-based, polyphenolic compound occurring naturally in nuts, berries, and the skin of grapes, and is present in many food items, particularly red wine [1]. In recent years, the clinical effects of resveratrol have been studied intensively owing to a plethora of promising effects in cells and animals; among these are improvement in terms of the various consequences of obesity, e.g., impaired insulin sensitivity and low-grade inflammation [2,3,4]. A landmark study demonstrated a shortened life span in diet-induced obese mice and a resveratrol-mediated improvement in longevity despite obesity [5]. Owing to its purported salutary effects, resveratrol is already being sold as an over-the-counter dietary supplement. Unfortunately, translating the effects of resveratrol to a human setting has proven difficult. Some clinical studies have confirmed the pre-clinical results of resveratrol-mediated improved insulin sensitivity, diminished low-grade inflammation, reduced blood pressure, and reduced amounts of liver fat accumulation [6,7]. In contrast, other studies fail to detect any positive physiological effects [8,9]. These conflicting results may in part be attributable to differences in study designs, populations, and resveratrol formulations. Some studies have been performed in healthy individuals, and the ability of resveratrol to improve glucose handling in situations with normal glucose homoeostasis has been questioned [8]. In contrast, it is feasible that the effect of a food ingredient is too weak to demonstrate any measurable consequences in full-blown type 2 diabetes. Thus, subjects suffering from modest degrees of insulin resistance are most likely optimal for the purpose of investigating the possible effects of resveratrol on insulin sensitivity, and probably most comparable to the original mice studies using diet-induced obese mice. Study length may also be of importance. Most studies include intervention periods of only a few weeks [6,9]. Obviously, longer studies provide a better determination of the possible effects of resveratrol on chronic conditions such as insulin resistance and low-grade inflammation. Finally, the methods currently used in most studies on resveratrol are targeted towards whole-body effects. Insulin sensitivity, for instance, has been assessed via measurements of specific targets such as plasma insulin, glucose, and HbA1c [6,8,9]. These techniques have their strengths, such as being very specific and applicable in daily clinics; however, the drawback is that findings will be limited to the pre-specified objectives. Likewise, when confining assessment to plasma samples, intracellular effects may be missed. 

Recently, the so-called metabolomics approach has been developed. Metabolomic analysis profiles small-molecule metabolites using either a targeted or non-targeted approach [10]. Through systematic identification and quantification of metabolites in biological systems, e.g. tissues, organs, or fluids at a certain time point, metabolomics directly sample metabolic modulations. Targeted metabolomics allows for very precise measurement of a specific target or an isolated group of related metabolites, whereas non-targeted metabolomics assesses as wide a range of different metabolites as possible. This technique provides a simultaneous screening of multiple metabolic pathways and captures an instantaneous integrated snapshot of the entire physiology of an organism. Metabolomics has the advantage over other “omics” that it integrates changes in gene expression, protein levels, enzymatic activity, and post-translational changes. Previously, metabolomics made it possible to determine changes in the human plasma metabolome caused by various conditions and diseases like sleep restriction [11,12], diabetes [13], and bariatric surgery [14].

Studies have demonstrated a weak correlation between plasma and muscle metabolite levels, indicating that plasma measurements are poor indicators of skeletal muscle metabolism [15]. Consequently, metabolomic analysis requires tissue biopsies to allow a detailed examination of changes in the intracellular pathways within this specific tissue. Such metabolic profiling offers the possibility to identify biochemical signatures of cellular metabolism involved in different pathways like glucose, lipid, and protein handling.

Massive research interest has gone into resveratrol as a potential protective compound in the management of obesity-associated complications, e.g., metabolic syndrome and low-grade inflammation. However, the particular pathways involved in potential salutary effects are by and large undetermined. Non-targeted metabolomic profiling may provide new insight into these matters. We have recently conducted a four-month randomized, placebo-controlled clinical trial describing the effects of resveratrol treatment in middle-aged males with metabolic syndrome and reported the findings on bone [16], circulating steroids [17], and glucose metabolism and inflammation [18]. The aim of the present study is to provide a comprehensive metabolomic analysis of the changes caused by four months of high-dose resveratrol treatment in these middle-aged men with metabolic syndrome. As the effects of resveratrol in humans are incompletely characterized, we used a non-targeted metabolomics approach based on four different matrices: plasma, urine, adipose tissue, and skeletal muscle tissue from each study subject. In our study on circulating steroids and prostate size we established a dose-dependent decrease in plasma androgen precursors by resveratrol [17]. In order to maximize the chances of detecting even subtle resveratrol-induced pathway changes, we therefore decided to employ the metabolomics analysis in the high-dose resveratrol (hRSV) (500 mg twice daily) group compared to the placebo group.

## 2. Results

### 2.1. Clinical Features

The basic characteristics of the participants are outlined in Table 1. Apart from a slight difference in age, the two groups were comparable at baseline and post-treatment.

### 2.2. Global Metabolic Profiling

**Figure 1:**
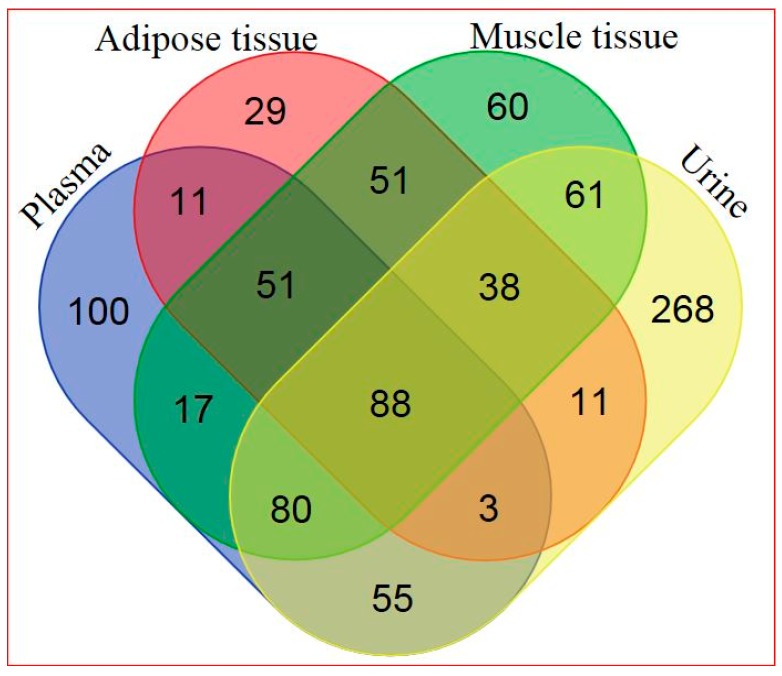
Venn diagram of all detected metabolites in the four different matrices (for metabolite details see Supplementary Table S5).

**Figure 2:**
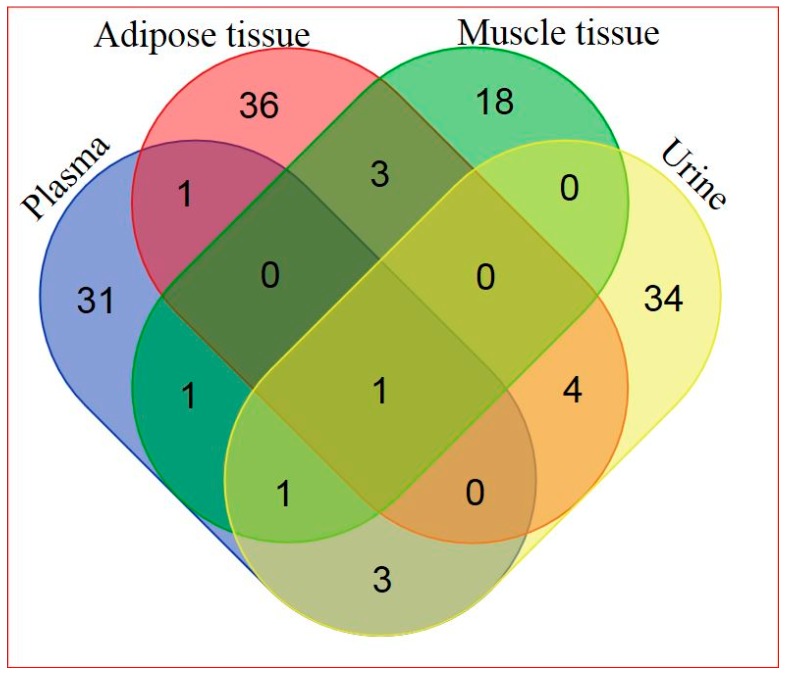
Venn diagram of significantly hRSV-altered metabolites detected in the four different matrices (for metabolite details see Supplementary Table S6).

Based on a combination of two Ultrahigh Performance Liquid Chromatography-Tandem Mass Spectroscopy (UPLC-MS/MS) platforms and one Gas Chromatography–Mass Spectroscopy (GS-MS) platform, 405 metabolites in plasma, 282 metabolites in adipose tissue, 446 metabolites in skeletal muscle, and 604 metabolites in urine were identified. General platform methods, data analysis, and metabolite detection identification are described in detail in the Supplementary Materials (see Supplementary Appendix and Supplementary Tables S1–S4). A total of 88 identified metabolites were present in all four matrices, as illustrated in Figure 1. The relationship between significantly hRSV-changed metabolites in the four matrices is depicted in Figure 2. Adipose tissue and urine had the greatest overlap in terms of significantly changed metabolites. One metabolite, dehydroisoandrosterone sulfate (DHEA-S), was significantly changed in all four matrices.

Random forest analysis (RF analysis) of adipose tissue, muscle tissue, urine, and plasma metabolic profiles resulted in 75%, 87%, 82%, and 89% accuracy in differentiating the hRSV and placebo groups, respectively, indicating that the differences in biochemical profiles between the two groups were quite pronounced. Random Forest classification results, methods, and data analysis are described in detail in the Supplementary Materials (see Supplementary Appendix and Supplementary Table S7). Among the 30 top-ranking metabolites resulting from the RF analysis, biochemicals involved in amino acid metabolism and lipid metabolism were particularly important with regards to the separation of the two groups. Many of the metabolites embraced in the RF-analysis are also included in the hRSV-altered metabolic pathways. Steroid hormones, especially, were well represented in the RF importance plots, in addition to being significantly altered by hRSV intervention.

### 2.3. Metabolic Profiling in Adipose Tissue

**Figure 3:**
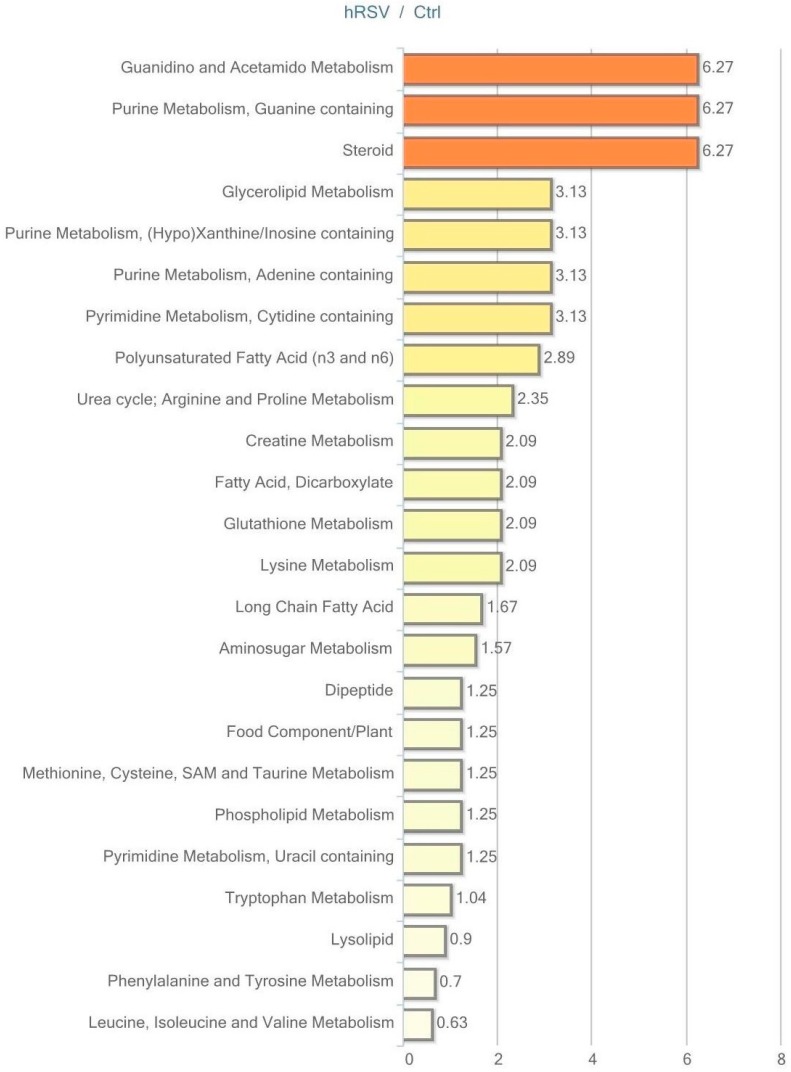
Resveratrol-mediated changes in adipose tissue, expressed as Pathway enrichment values (PEV). Values are based on the significant resveratrol-regulated compounds relative to all detected compounds in the pathway. PEV = (number of significant metabolites in pathway/total number of detected metabolites in pathway)/(total number of significant metabolites/total number of detected metabolites); hRSV: high-dose resveratrol; Ctrl: Placebo group.

Of the 282 biochemicals identified in adipose, tissue 45 displayed a significant change (*p* ≤ 0.05) in response to hRSV treatment: 31 of these were elevated and 14 were reduced. Furthermore, for 28 compounds there was a trend towards a change (*p* < 0.10), of which 24 compounds were increased and four compounds were reduced by hRSV treatment. The pathways that differed significantly between the hRSV group and controls in adipose tissue (*p* < 0.05) are shown in Figure 3.

Exploring the respective significantly changed pathways in detail reveals the specific hRSV-affected compounds. As illustrated in Table 2, four out of 15 identified lipids are significantly elevated in the long-chain fatty acid pathway (myristate (14:0), myristoleate (14:1n5), palmitate (16:0), and palmitoleate (16:1n7)). These lipids depict the significant fold change between the hRSV group and the placebo group. In addition, examining the polyunsaturated fatty acid pathway demonstrated that six out of 13 identified lipids are significantly elevated in the hRSV group (stearidonate (18:4n3), docosapentaenoate (n3 DPA; 22:5n3), docosahexaenoate (DHA; 22:6n3) linoleate (18:2n6) adrenate (22:4n6), and mead acid (20:3n9)). Also of interest, the steroid hormone pathway reveals significant reductions in both of the identified steroids, dehydroisoandrosterone sulfate (DHEA-S) and 4-androsten-3β, 17β-diol disulfate.

The most interesting trending changes (*p* < 0.10) in adipose tissue pathways were displayed in the glycolysis, gluconeogenesis, and pyruvate metabolism pathway and the pentose phosphate pathway. As illustrated in Table 2, trending elevation in three out of nine identified glycolytic pathway intermediates (glucose-6-phosphate (G6P), dihydroxyacetone phosphate (DHAP), 3-phosphoglycerate, phosphoenolpyruvate (PEP)), and one in the pentose phosphate pathway intermediate was found (sedoheptulose-7-phosphate).

### 2.4. Metabolic Profiling in Plasma

**Figure 4:**
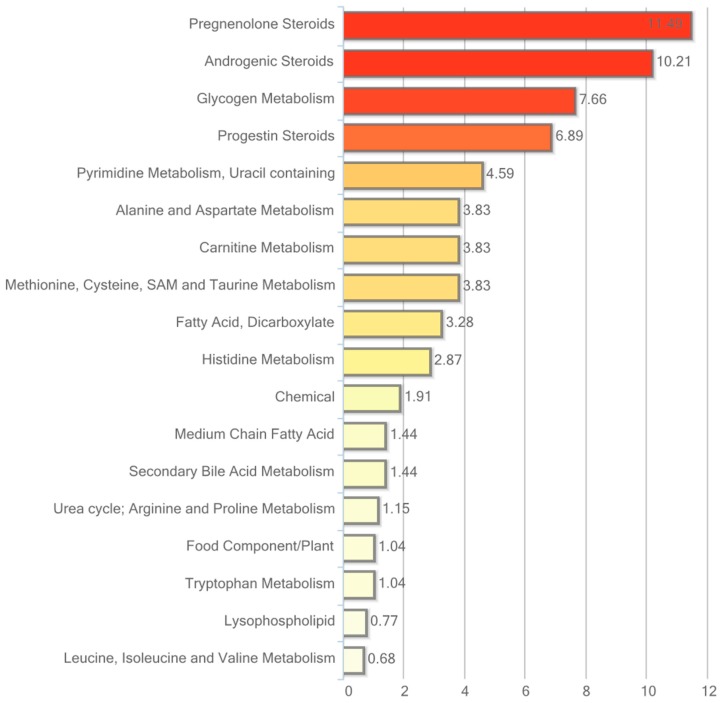
Resveratrol-mediated changes in plasma expressed as Pathway enrichment values (PEV). Values are based on the significant resveratrol-regulated compounds relative to all detected compounds in the pathway. PEV = (number of significant metabolites in pathway/total number of detected metabolites in pathway)/(total number of significant metabolites/total number of detected metabolites).

Of the 405 named compounds in plasma, 38 were statistically different between the two groups (*p ≤* 0.05); 13 of these were elevated and 25 were reduced. In addition, 11 compounds showed a trend towards a change by hRSV (*p* < 0.10), of which eight were increased and three were reduced. The pathways that are significantly different (*p* < 0.05) between the hRSV group and controls are shown in Figure 4.

Particularly interesting changes in response to hRSV treatment were found in the steroid hormone pathway with consistent significant reductions in circulating levels of cholesterol-derived steroid hormones and sulfated steroid hormones. As illustrated in Table 3, 13 out of 19 detected steroid hormones were significantly reduced.

Also noteworthy, significant elevations in histidine and the related biochemical marker of muscle protein turnover 3-methylhistidine along with its acetylated derivative *N*-acetyl-3-methylhistidine were observed in plasma in response to hRSV treatment. In addition, subtle but consistent reductions in the dicarboxylic acids dodecanedioate, tetradecanedioate, sebacate, hexadecanedioate, and octadecanedioate were observed in the hRSV group.

### 2.5. Metabolic Profiling in Skeletal Muscle Tissue

**Figure 5:**
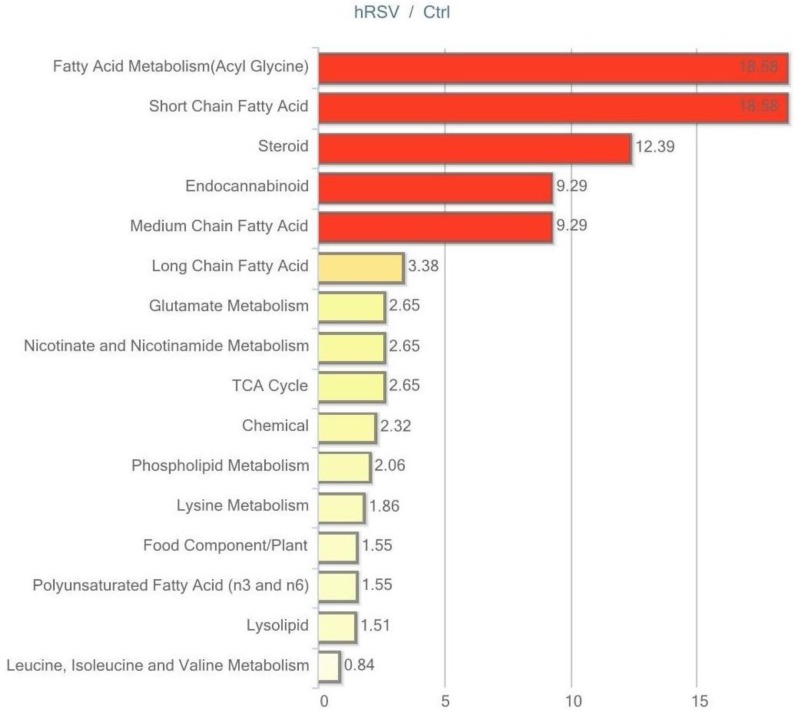
Resveratrol-mediated changes in skeletal muscle tissue expressed as Pathway enrichment values (PEV). Values are based on the significant resveratrol-regulated compounds relative to all detected compounds in the pathway. PEV = (number of significant metabolites in pathway/total number of detected metabolites in pathway)/(total number of significant metabolites/total number of detected metabolites); hRSV: high-dose resveratrol; Ctrl: Placebo group.

Of the 446 named biochemicals in skeletal muscle tissue, 24 changed significantly (*p* ≤ 0.05); 12 of these were elevated and 12 reduced by hRSV treatment. Additionally, 19 biochemicals showed a trend towards a change by hRSV (*p* < 0.10), with 12 biochemicals increased and seven reduced. The pathways that are significantly different (*p* < 0.05) between the hRSV group and controls are shown in Figure 5.

The most striking change in muscle tissue was the effect of hRSV on the steroid hormone pathway, with four out of six compounds being significantly reduced, as illustrated in Table 4. All four compounds comprised sulfated steroid metabolites (dehydroisoandrosterone sulfate (DHEA-S), epiandrosterone sulfate, androsterone sulfate, 4-androsten-3β, and 17β-diol disulfate 1).

Regarding skeletal muscle lipid metabolism, our analysis revealed significant hRSV alterations in the short-chain fatty acids, medium-chain fatty acid, and long-chain fatty acid pathways. Generally, the hRSV group had lower levels of intracellular lipids. In addition, two out of 12 biochemicals in the polyunsaturated fatty acid pathway were increased (stearidonate (18:4n3) and linolenate [α or γ; (18:3n3 or 6)]).

### 2.6. Metabolic Profiling in Urine

**Figure 6:**
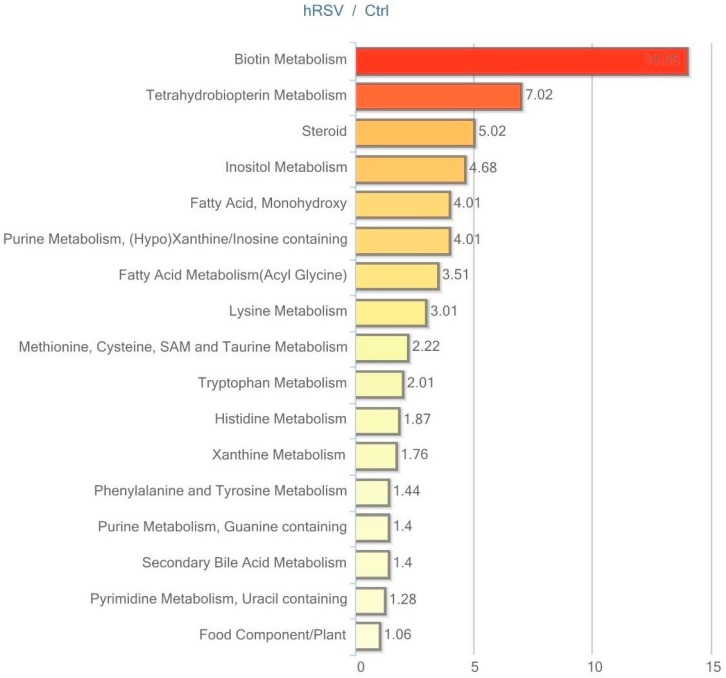
Resveratrol-mediated changes in urine expressed as Pathway enrichment values (PEV). Values are based on the significant resveratrol-regulated compounds relative to all detected compounds in the pathway. PEV = (number of significant metabolites in pathway/total number of detected metabolites in pathway)/(total number of significant metabolites/total number of detected metabolites); hRSV: high-dose resveratrol; Ctrl: Placebo group.

Urine data were analyzed in both non-normalized and osmolality-normalized formats, and because normalization to osmolality values did not substantially affect results, non-normalized data were utilized for data interpretation. Of the 604 named biochemicals in urine, 43 changed significantly (*p* ≤ 0.05); 31 of these were elevated and 12 were reduced by hRSV treatment. Additionally, 27 biochemicals showed a trend towards a change by hRSV (*p* < 0.10), with 22 biochemicals increased and five reduced. Pathways that demonstrated significantly modifications (*p* < 0.05) in response to hRSV are shown in Figure 6.

The most striking hRSV-induced changes in urine were observed in the steroid hormone pathway. Ten out of 28 named steroids were significantly changed, with the majority being elevated, as illustrated in Table 5.

Furthermore, several metabolites derived from aromatic amino acids that have a contribution from or are exclusively produced by the gut microbiota were altered in the urine. The tyrosine-derived metabolites tyramine, phenol sulfate and homovanillate (HVA), were changed in the hRSV group. Similarly, urinary levels of derivatives from the tryptophan metabolism (tryptamine and indolelactate), the phenyalanine metabolism (2-hydroxyphenylacetate), and the histidine metabolism (imidazole propionate) were significantly altered by hRSV treatment, as depicted in Table 5.

## 3. Discussion

The effects of resveratrol in human trials have proven to be very inconsistent. Most data represent standard biochemical analyses of selected parameters. The focus has been on inflammation [8,19], glucose metabolism [9,20], lipids [21], liver function [22], and steroids [17], assessed by measurement in blood samples, thus representing a picture of whole body metabolism. The present paper describes an exhaustive metabolomic profile of blood, adipose tissue, skeletal muscle tissue, and urine from a comprehensive clinical trial of high-dose resveratrol-treated, middle-aged males with metabolic syndrome. This approach allows for a more elaborate investigation of subtle resveratrol effects on distinct intracellular pathways than what is possible by measurements confined to blood. In addition, this approach of unbiased measurements in various body compartments may help us to determine the cause of some of the effects we have previously detected. Recently, we reported that the androgen precursors in blood were significantly reduced after resveratrol treatment for four months [17]. The presented metabolomic analysis was performed in the same study cohort, and by using this advanced technique we are able to verify the decrease in sulfated androgen precursors in the blood [17]. In addition, we find significantly lower intracellular amounts of sulfated androgen precursors in skeletal muscle tissue as well as adipose tissue. Sulfation is a common modification to control levels of active steroid hormones at target sites by increasing solubility and renal excretion. The metabolomic profiling of urine revealed increased urinary excretion of the majority of the measured sulfated steroid hormones. In the original paper on reduced androgen precursors, we discussed whether the reduction was caused by decreased formation or increased excretion. The present results propose that at least part of the decrease in sulfated androgen precursors in blood and tissues is caused by an increase in urinary excretion of the sulfated steroids.

The metabolomic analysis of lipids points to a striking accumulation of long-chain saturated, monounsaturated, and polyunsaturated (n3 and n6) free fatty acids in adipose tissue. However, in skeletal muscle two long-chain lipids were significantly changed; one was elevated (10-heptadecenoate (17:1n7)) whereas the other was significantly reduced (arachidate (20:0)). Deducing how resveratrol induces the increase in many of the long-chain polyunsaturated fatty acids (n3 and n6) in adipose tissue is complex. Usually these long-chain polyunsaturated fatty acids derive from increased intake of seafood or special plant oils. However, the two essential fatty acids alpha-linolenic acid (ALA; 18:3n3) and linoleic acid (LA; 18:2n6) can be metabolized into longer-chain polyunsaturated fatty acids through a series of desaturation and elongation processes [23]. The literature describes a gender difference in the conversion of ALA to eicosapentaenoic acid (EPA) and docosahexaenoic acid (DHA), with women having a much higher conversion rate than men [24,25]. This difference depends upon sex steroids, and testosterone supplementation to female transsexuals reduced DHA concentration by 22% [26]. Clearly, this indicates that the enzymes responsible for the desaturation and elongation conversion of ALA to DHA are negatively regulated by androgens. Based on this knowledge, we speculate that resveratrol might stimulate the conversion process of ALA and LA, resulting in an increase in long-chain polyunsaturated fatty acids through the robust reduction in androgen precursors.

The health impacts of changes in levels of long-chain polyunsaturated fatty acids are still not fully determined. Long-chain polyunsaturated fatty acids have been associated with a positive effect on insulin signaling via the insulin receptor [27], and even a minor increase of ALA content in adipose tissue of 1% has been associated with a 5 mmHg decrease in blood pressure [28]. However, intervention studies providing ALA have yielded conflicting results on blood pressure, cholesterol levels, and triglyceride levels, as reviewed by Baker et al. [23]. Also, despite the increase in these fatty acids in adipose tissue after resveratrol treatment, no clinically relevant improvements in blood pressure or insulin sensitivity were observed in our study population [18].

In the adipose tissue we also detected a significant increase in intracellular glycerol and free fatty acids in the resveratrol-treated subjects. In support of this notion, previously published cell culture studies revealed a resveratrol-mediated enhancement of adrenergic stimulated lipolysis in fat cells [29,30].

Moreover, resveratrol treatment caused consistent reductions in several dicarboxylic fatty acids in plasma. These lipids can be produced from peroxisomal-mediated degradation of very long chain fatty acids (>22 carbons) and subsequently be metabolized via beta-oxidation. Collectively, a decrease in the dicarboxylic fatty acids in plasma could be a result of increased beta-oxidation in adipose tissue.

Regarding glucose metabolism, we found trends toward elevation in glycolytic intermediates and the pentose phosphate pathway (PPP) intermediates (*p* < 0.10) in adipose tissue in response to resveratrol. Stimulation of the PPP pathway is associated with increased insulin resistance, increased NADPH production, and thus, increased production of free fatty acids [31]. Hence, our metabolomics findings may indicate increased glucose availability and utilization through glycolysis and the pentose phosphate pathway, owing to decreased insulin sensitivity in the males receiving resveratrol. Clinical parameters in the same study subjects support this assumption, as resveratrol increased fructosamine levels after four months of treatment, indicating poorer insulin sensitivity [18].

In the resveratrol-treated group we demonstrated elevation in metabolites related to metabolism of the amino acid histidine. In particular, histidine was significantly increased in the blood and a trending elevation in the related biochemical marker of the muscle protein turnover 3-methylhistidine along with the acetylated derivative *N*-acetyl-3-methylhistidine was also observed. Increase in 3-methylhistidine, which is a post-translationally modified amino acid derived from the muscle contractile proteins actin and myosin, and *N*-acetyl-3-methylhistidine indicates increased muscle turnover in men treated with resveratrol. This finding ties in well with the study by Olesen et al. [32], in which resveratrol blunted the positive effects of exercise training. In contrast, Scribbans et al. found that resveratrol in addition to exercise had positive effects on muscle metabolism with an increase in mitochondrial capacity [33], while others found no effect in humans [34]. Most rodent studies have found positive effects of resveratrol on muscle function [5,35,36]. Clearly, more clinical studies are needed to determine the exact effects of resveratrol on skeletal muscle function.

Finally, we found urinary changes in several tyrosine-derived, tryptophan-derived, phenylalanine-derived, and histidine-derived metabolites. Tryptophan, histidine, and phenylalanine are classified as essential amino acids, which cannot be synthesized de novo by the body. However, gut bacteria may contribute to their production and degradation [37]. Fermentation of tyrosine and tryptophan by colonic bacteria is found to produce phenols and indoles, which are excreted in the urine [38]. Also, imidazole propionate is known to be excreted in the urine and direct degradation of urocanate to imidazole propionate by the intestinal flora was demonstrated in an animal study [39]. Changes in microbiota-related urinary metabolites are proposed to be of relevance to human health. For instance hippurate, phenylacetylglutamine, 4-cresylsulfate, and 4-hydroxyphenylpropionate have been related to body weight, blood pressure, and metabolic syndrome [40], while urinary imiadazole propionate, which we found to be elevated by resveratrol, has been related to intestinal dysfunction [39]. Therefore, the changes in urinary amino acids and derivatives revealed by the metabolomic analysis may imply a resveratrol-induced modulation of the gut flora in men with metabolic syndrome. This is in accordance with studies in rodents where resveratrol has been shown to modulate the gut microbiota directly [41,42,43].

The strengths of the present clinical trial are a carefully selected, strong study design and the advanced metabolomics approach. A high dosage of resveratrol in a relatively long-term study was chosen. Participants were selected because they had metabolic syndrome, a condition considered a prime target for resveratrol. Furthermore, the advanced metabolomics technique was applied in an unbiased approach to four different matrices. However, a limitation of our study is the lack of liver tissue samples. The liver is a highly metabolic organ and a number of experimental studies have provided evidence of the hepatic benefits of resveratrol treatment [44]. Metabolomic profiling of liver tissue from the same study subjects would help complete the metabolic puzzle of resveratrol-induced biochemical effects. Also, metabolomic analysis on feces would have provided insight into possible beneficial effects on the gut microbiota. Finally, it must be kept in mind that the present study used a very high dose of resveratrol (1000 mg), which is impossible to reach from natural food items. Resveratrol has a very low bioavailability owing to a rapid metabolism to glucuronide and sulfate conjugates in the liver. However, it is possible that ingestion of food items rich in resveratrol and other polyphenols might inhibit the metabolism or increase the absorption of resveratrol, resulting in a better bioavailability from food items than from purified resveratrol products [45,46].

## 4. Materials and Methods

### 4.1. Study Design

Data in the present study comprise a subgroup from our original study describing the effects of resveratrol on bone [16]. As we only obtained tissue samples from 24 subjects in the placebo group and 21 in the hRSV group, the sample size is lower compared to the original paper. The protocol was approved by the Regional Committee on Health Research Ethics (M-20110111) (24 May 2011) and the Danish Data Protection Agency, and the study was conducted in agreement with the Declaration of Helsinki II. Participants were given oral and written information about the purpose and nature of all procedures before informed consent was obtained. The protocol was registered at clinicaltrials.gov (NCT01412645).

The study was a randomized, placebo-controlled, double-blinded, single-center study. Male test subjects with metabolic syndrome (MetS) were randomized to treatment for four months with tablets containing a placebo, low-dose resveratrol (75 mg twice daily), or high-dose resveratrol (500 mg twice daily). The study comprised 66 test subjects. Inclusion criteria were: Male gender, age between 30 and 60 years, and MetS. MetS was defined according to the International Diabetes Federation [47] as central obesity (Waist circumference ≥94 cm and/or BMI > 30 kg/m^2^) plus any two of the following: raised triglycerides (≥1.7 mmol/L), reduced high-density lipoprotein (HDL) (≤1.03 mmol/L), raised blood pressure (systolic ≥130 mmHg or diastolic ≥85 mmHg), raised fasting plasma glucose (≥5.6 mmol/L), or drug treatment for the individual features. The trial was performed under conditions of weight stability, unchanged diet, unchanged dietary supplements, and strict compliance with the study drug. Metabolomics results from the high-dose resveratrol (1000 mg daily) and the placebo groups after four months’ treatment are reported in the present study.

### 4.2. Samples

Blood and urine samples were collected between 7:30 and 11:00 a.m. after an overnight fast. Biopsy of the abdominal subcutaneous adipose tissue depot was obtained using a liposuction cannula and skeletal muscle biopsy was harvested from the musculus vastus lateralis using a Bergström cannula. All procedures were performed under sterile conditions. Before both biopsies were performed, the area was anesthetized using 5 to 10 mL lidocaine.

### 4.3. Metabolomic Analysis

Urine, blood, adipose tissue, and skeletal muscle tissue samples were shipped to Metabolon, Inc.^®^, (Durham, NC, USA) on dry ice. Following receipt, samples were stored at −80 °C until analyzed. A recovery standard was added prior to the first step in preparation for quality control (QC). To remove protein, dissociate small molecules bound to protein or trapped in the protein matrix, and to recover chemically diverse metabolites, proteins were precipitated with methanol under vigorous shaking for two minutes followed by centrifugation. The resulting extracts were divided into five fractions: one for analysis by Ultrahigh Performance Liquid Chromatography-Tandem Mass Spectroscopy (UPLC-MS/MS) with positive ion mode electrospray ionization, one for analysis by UPLC-MS/MS with negative ion mode electrospray ionization, one for LC polar platform, one for analysis by Gas Chromatography-Mass Spectroscopy (GC-MS), and one sample was reserved for backup. Raw data were extracted, peak-identified, and processed using Metabolon’s hardware and software. More than 3300 commercially available purified standard compounds were used for the identification of compounds in the samples. General platform methods and metabolite detection identification are described in detail in the Supplementary Materials (see Supplementary Appendix, Tables S8 and S9, Figures S1 and S2, and Supplementary Tables S1–S4).

### 4.4. Statistical Analysis

Baseline comparisons of the study population were evaluated by unpaired Student’s *t*-test after being checked for normality and equal variance using SPSS Statistics version 20 software. Results are expressed as mean ± SEM. The level of significance was 0.05.

The pathway enrichment value (PEV) used in Figure 3, Figure 4, Figure 5 and Figure 6 is calculated based on the following equation:

PEV = (number of significant metabolites in pathway/total number of detected metabolites in pathway)/(total number of significant metabolites/total number of detected metabolites)(1)

A PEV greater than one indicates that the pathway holds more hRSV-regulated compounds relative to the study overall, suggesting that the pathway may be a target of interest in the intervention effects.

In the metabolomic analysis Welch’s two-sample *t*-test was used to identify biochemicals that differed significantly between treatment groups, following log transformation and, if necessary, imputation of missing values with the minimum observed value for each compound. *p*-Values ≤ 0.05 were considered statistically significant and values approaching significance (*p* < 0.10) were reported as well. An estimate of the false discovery rate (*q*-value method) was calculated to take into account the multiple comparisons that normally occur in metabolomics-based studies. Metabolomics statistical methods are described in details in the Supplementary Materials (see Supplementary Appendix and Table S10).

## 5. Conclusions

Our comprehensive metabolomic analysis revealed small but robust changes in response to high-dose resveratrol treatment. The main finding is that resveratrol lowered sulfated androgen precursors in blood, adipose tissue, and muscle tissue and concurrently increased the content in urine, indicating increased urinary excretion of these sulfated steroids. Furthermore, the content of long-chain polyunsaturated fatty acids in adipose tissue was increased, probably by increased conversion of ALA and LA mediated through reduced androgen precursors. Several metabolites derived from aromatic amino acids associated with the gut microbiota changed, suggesting that resveratrol may affect either the composition or the metabolism of the gut flora. Considering the increasingly recognized role of the gut microbiota in human health and disease, this effect on gut flora activity may represent a potential and import mode of action of resveratrol. Lastly, our results indicate that non-targeted metabolomics is able to broaden our understanding of the many intra-cellular processes regulated by resveratrol treatment. Yet, our study also demonstrates that it is difficult to conclude solely from a single matric, since only a minor overlap between the four matrices we examined was found. Based on this, we suggest that metabolomics are performed in the tissue of interest. Future clinical studies on resveratrol should focus on the steroid metabolism pathway and the direct impact on human gut microbiota, preferably including liver tissue and feces samples.

## Figures and Tables

**Table 1:** Clinical features at baseline and post-treatment.

Characteristic	Placebo (*n* = 24)		hRSV (*n* = 21)		*p*-Value
	Baseline	4 Months	Baseline	4 Months	
Age, years	47.8 ± 1.3		51.9 ± 1.3		*p* < 0.05
Body mass index, kg/m^2^	34.1 ± 0.8	33.4 ± 0.2	33.8 ± 0.7	33.7 ± 0.2	NS
HOMA-IR	4.36 ± 0.5	4.19 ± 0.3	3.87 ± 0.5	4.50 ± 0.3	NS
Systolic blood pressure, mmHg	150 ± 3	142 ± 3	146 ± 2	140 ± 3	NS
Diastolic blood pressure, mmHg	91.3 ± 2.1	86.0 ± 1.3	89.3 ± 1.7	87.8 ± 1.4	NS

^1^ Data are expressed as mean ± SEM. NS = no significant difference. Comparisons between groups were evaluated by unpaired Student’s *t*-test. The level of significance was 0.05.

**Table 2:** Summary of resveratrol-induced changes in adipose tissue. Identified metabolites in the long-chain fatty acid, polyunsaturated fatty acid (n3 and n6), steroids, glycolysis, gluconeogenesis, and pyruvate metabolism, and pentose phosphate pathways in adipose tissue. Red: Significant elevation (*p* ≤ 0.05). Orange: Trending elevation (*p* < 0.10); Green: Significant reduction (*p* ≤ 0.05).

Superpathway	Sub Pathway	Biochemical Name	hRSV Ctrl	*p*-Value	*q*-Value
**Lipid**	Long-Chain Fatty Acid	Myristate (14:0)	1.10	0.003	0.111
		Myristoleate (14:1n5)	1.67	0.017	0.182
		Pentadecanoate (15:0)	1.01	0.977	0.816
		Palmitate (16:0)	1.10	0.033	0.240
		Palmitoleate (16:1n7)	1.41	0.003	0.109
		Margarate (17:0)	0.98	0.500	0.672
		10-heptadecenoate (17:1n7)	1.12	0.086	0.309
		Stearate (18:0)	1.02	0.610	0.731
		Oleate (18:1n9)	1.17	0.109	0.328
		*cis*-vaccenate (18:1n7)	1.17	0.304	0.538
		Nonadecanoate (19:0)	1.00	0.866	0.794
		10-nonadecenoate (19:1n9)	1.08	0.569	0.703
		Arachidate (20:0)	0.94	0.472	0.660
		Eicosenoate (20:1n9 or 11)	1.12	0.335	0.566
		Erucate (22:1n9)	0.96	0.657	0.734
	Polyunsaturated Fatty Acid (n3 and n6)	Stearidonate (18:4n3)	1.50	0.045	0.240
		Eicosapentaenoate (EPA; 20:5n3)	1.25	0.093	0.311
		Docosapentaenoate (n3 DPA; 22:5n3)	1.31	0.012	0.177
		Docosahexaenoate (DHA; 22:6n3)	1.38	0.042	0.240
		Linoleate (18:2n6)	1.24	0.021	0.203
		Linolenate [α or γ; (18:3n3 or 6)]	1.21	0.060	0.253
		Dihomo-linolenate (20:3n3 or n6)	1.19	0.065	0.261
		Arachidonate (20:4n6)	1.17	0.107	0.328
		Adrenate (22:4n6)	1.69	0.010	0.177
		Docosapentaenoate (n6 DPA; 22:5n6)	1.63	0.056	0.253
		Docosadienoate (22:2n6)	1.10	0.275	0.498
		Dihomo-linoleate (20:2n6)	1.11	0.216	0.443
		Mead acid (20:3n9)	1.33	0.045	0.240
	Steroid	Dehydroisoandrosterone sulfate (DHEA-S)	0.40	0.013	0.177
		4-androsten-3β,17β-diol disulfate (1)	0.37	0.009	0.177
**Carbohydrate**	Glycolysis, Gluconeogenesis, and pyruvate metabolism pathway	1,5-anhydroglucitol (1,5-AG)	0.86	0.235	0.464
		Glucose	0.98	0.594	0.722
		Glucose-6-phosphate (G6P)	1.23	0.059	0.253
		Isobar: fructose 1,6-diphosphate, glucose 1,6-diphosphate, myo-inositol 1,4 or 1,3-Diphosphate	1.16	0.159	0.394
		Dihydroxyacetone phosphate (DHAP)	1.30	0.080	0.303
		3-phosphoglycerate	1.75	0.057	0.253
		Phosphoenolpyruvate (PEP)	1.39	0.128	0.357
		Lactate	1.04	0.396	0.599
		Glycerate	1.08	0.771	0.777
	Pentose phosphate pathway	Sedoheptulose-7-phosphate	1.27	0.051	0.253

**Table 3:** Summary of resveratrol-induced changes in plasma. Identified metabolites in the steroid, fatty acid dicarboxylate, and histidine metabolism pathways in plasma. Red: Significant elevation (*p* ≤ 0.05). Orange: Trending elevation (*p* < 0.10); Green: Significant reduction (*p* ≤ 0.05). Light green: Trending reduction (*p* < 0.10).

Superpathway	Sub Pathway	Biochemical Name	hRSV Ctrl	*p*-Value	*q*-Value
**Lipid**	Steroid	Pregnenolone sulfate	0.63	0.001	0.018
		21-hydroxypregnenolone disulfate	0.41	<0.001	<0.001
		5α-pregnan-3β,20α-diol disulfate	0.42	<0.001	<0.001
		5α-pregnan-3α,20β-diol disulfate 1	0.93	0.140	0.762
		Pregnen-diol disulfate	0.53	0.001	0.015
		Pregn steroid monosulfate	0.72	0.046	0.515
		Pregnanediol-3-glucuronide	0.92	0.644	0.927
		Cortisol	0.92	0.745	0.941
		Cortisone	0.93	0.285	0.824
		Dehydroisoandrosterone sulfate (DHEA-S)	0.57	<0.001	0.013
		Epiandrosterone sulfate	0.42	<0.001	0.004
		Androsterone sulfate	0.40	<0.001	0.004
		4-androsten-3β,17β-diol disulfate 1	0.51	<0.001	0.016
		4-androsten-3β,17β-diol disulfate 2	0.49	<0.001	<0.001
		5α-androstan-3β,17α-diol disulfate	0.37	<0.001	0.001
		5α-androstan-3α,17β-diol disulfate	0.71	0.071	0.659
		5α-androstan-3β,17β-diol disulfate	0.37	<0.001	0.002
		Andro steroid monosulfate 2	0.31	<0.001	<0.001
		Estrone 3-sulfate	0.87	0.132	0.762
	Fatty Acid, Dicarboxylate	2-hydroxyglutarate	0.98	0.660	0.934
		Sebacate (decanedioate)	0.73	0.101	0.762
		Dodecanedioate	0.61	<0.001	0.001
		Tetradecanedioate	0.75	0.021	0.278
		Hexadecanedioate	0.85	0.206	0.762
		Octadecanedioate	0.88	0.255	0.811
		3-carboxy-4-methyl-5-propyl-2-furanpropanoate (CMPF)	0.95	0.955	0.964
**Amino Acid**	Histidine Metabolism	Histidine	1.16	0.008	0.147
		3-methylhistidine	1.93	0.056	0.570
		*N*-acetyl-3-methylhistidine	1.23	0.084	0.721
		*trans*-urocanate	1.38	0.128	0.762

**Table 4:** Summary of resveratrol-induced changes in skeletal muscle tissue. Identified metabolites in the steroid, short-chain fatty acid, medium-chain fatty acid, long-chain fatty acid, and polyunsaturated fatty acid (n3 and n6) pathways in skeletal muscle. Red: Significant elevation (*p* ≤ 0.05). Orange: Trending elevation (*p* < 0.10); Green: Significant reduction (*p* ≤ 0.05).

Superpathway	Sub Pathway	Biochemical Name	hRSV Ctrl	*p*-Value	*q*-Value
**Lipid**	Steroid	Cortisol	1.40	0.211	0.962
		Cortisone	1.14	0.778	0.987
		Dehydroisoandrosterone sulfate (DHEA-S)	0.48	<0.001	0.025
		Epiandrosterone sulfate	0.29	<0.001	0.001
		Androsterone sulfate	0.24	<0.001	0.001
		4-androsten-3β,17β-diol disulfate (1)	0.46	0.002	0.125
	Short-Chain Fatty Acid	Valerate	0.86	<0.001	0.026
	Medium-Chain Fatty Acid	Caproate (6:0)	1.09	0.442	0.962
		Heptanoate (7:0)	0.82	<0.001	0.032
		Caprylate (8:0)	1.08	0.619	0.962
		Undecanoate (11:0)	0.89	0.002	0.125
	Long-Chain Fatty Acid	Myristoleate (14:1n5)	1.78	0.252	0.962
		Palmitate (16:0)	0.98	0.449	0.962
		Margarate (17:0)	0.95	0.268	0.962
		10-heptadecenoate (17:1n7)	1.09	0.029	0.655
		Stearate (18:0)	0.97	0.209	0.962
		Oleate (18:1n9)	1.01	0.994	0.996
		*cis*-vaccenate (18:1n7)	1.02	0.904	0.995
		10-nonadecenoate (19:1n9)	0.97	0.882	0.995
		Arachidate (20:0)	0.84	0.003	0.168
		Eicosenoate (20:1n9 or 11)	1.01	0.958	0.996
		Erucate (22:1n9)	0.88	0.193	0.962
	Polyunsaturated Fatty Acid (n3 and n6)	Stearidonate (18:4n3)	1.45	0.015	0.474
		Eicosapentaenoate (EPA; 20:5n3)	0.98	0.554	0.962
		Docosapentaenoate (n3 DPA; 22:5n3)	1.04	0.511	0.962
		Docosahexaenoate (DHA; 22:6n3)	1.07	0.344	0.962
		Linoleate (18:2n6)	1.02	0.714	0.980
		Linolenate [α or γ; (18:3n3 or 6)]	1.16	0.065	0.962
		Dihomo-linolenate (20:3n3 or n6)	1.02	0.873	0.995
		Arachidonate (20:4n6)	1.00	0.990	0.996
		Docosapentaenoate (n6 DPA; 22:5n6)	0.99	0.994	0.996
		Docosadienoate (22:2n6)	0.93	0.680	0.962
		Dihomo-linoleate (20:2n6)	0.83	0.287	0.962
		Mead acid (20:3n9)	0.93	0.619	0.962

**Table 5:** Summary of resveratrol-induced changes in urine. Identified metabolites in the steroid, tryptophan metabolism, phenylalanine and tyrosine metabolism, and histidine metabolism pathways in urine. Red: Significant elevation (*p* ≤ 0.05). Orange: Trending elevation (*p* < 0.10); Green: Significant reduction (*p* ≤ 0.05).

Superpathway	Sub Pathway	Biochemical Name	hRSV Ctrl	*p*-Value	*q*-Value
**Lipid**	Steroid	21-hydroxypregnenolone disulfate	0.50	0.012	0.313
		Pregnen-diol disulfate	0.79	0.421	0.861
		Pregn steroid monosulfate	2.10	0.008	0.281
		Pregnanediol-3-glucuronide	0.87	0.910	0.938
		Cortisol	1.29	0.734	0.915
		Cortisol glucuronide	1.36	0.600	0.893
		Cortisone	1.13	0.757	0.915
		Tetrahydrocortisone	1.54	0.270	0.762
		Dehydroisoandrosterone sulfate (DHEA-S)	3.95	<0.001	0.001
		16a-hydroxy DHEA 3-sulfate	1.04	0.201	0.735
		Epiandrosterone	0.87	0.967	0.938
		Epiandrosterone sulfate	1.43	0.036	0.575
		Androsterone sulfate	1.22	0.171	0.724
		4-androsten-3β,17β-diol monosulfate (1)	3.75	<0.001	0.001
		4-androsten-3β,17β-diol monosulfate (2)	3.50	0.003	0.149
		4-androsten-3α,17α-diol monosulfate (2)	6.78	<0.001	0.001
		4-androsten-3β,17β-diol disulfate (1)	0.72	0.804	0.920
		4-androsten-3β,17β-diol disulfate (2)	0.89	0.736	0.915
		5α-androstan-3β,17α-diol disulfate	0.20	0.002	0.127
		5α-androstan-3β,17β-diol disulfate	0.68	0.414	0.861
		Andro steroid monosulfate (1)	0.45	0.004	0.170
		Testosterone sulfate	3.74	0.009	0.281
		11-ketoetiocholanolone glucuronide	0.98	0.793	0.920
		Etiocholanolone glucuronide	0.85	0.766	0.915
		17α-hydroxypregnanolone glucuronide	1.10	0.216	0.747
		5β-pregnan-3α,21-diol-11,20-dione 21-Glucosiduronate	1.51	0.604	0.893
		11-ketoetiocholanolone sulfate	1.23	0.957	0.938
		Dehydroepiandrosterone glucuronide	0.83	0.585	0.881
**Amino Acid**	Tryptophan Metabolism	Tryptophan	0.99	0.908	0.938
		*N*-acetyltryptophan	1.37	0.194	0.726
		Tryptamine	0.37	<0.001	0.010
		Indolelactate	1.56	0.050	0.613
		Indoleacetate	0.58	0.201	0.735
		3-indoxyl sulfate	1.39	0.124	0.724
		Kynurenine	1.29	0.637	0.905
		Kynurenate	1.17	0.325	0.793
		Anthranilate	1.16	0.446	0.861
		3-hydroxykynurenine	1.43	0.480	0.874
		3-hydroxyanthranilate	1.59	0.057	0.638
		Xanthurenate	1.23	0.231	0.748
		Picolinate	1.16	0.180	0.724
		5-hydroxytryptophan	1.11	0.383	0.848
		5-hydroxyindoleacetate	1.02	0.563	0.881
		Serotonin (5HT)	1.03	0.782	0.920
		Indoleacetylglutamine	0.64	0.824	0.920
		Tryptophan betaine	1.14	0.976	0.940
		Indole-3-carboxylic acid	0.93	0.696	0.914
		*C*-glycosyltryptophan	1.03	0.693	0.914
		*N*-acetylkynurenine (2)	2.14	0.047	0.613
	Phenylalanine and Tyrosine Metabolism	Phenylalanine	1.11	0.588	0.881
		*N*-acetylphenylalanine	1.31	0.185	0.726
		Phenyllactate (PLA)	1.86	0.148	0.724
		4-hydroxyphenylacetate	1.02	0.915	0.938
		3-hydroxyphenylacetate	1.52	0.096	0.724
		Phenylacetylglycine	1.13	0.801	0.920
		Phenylacetylglutamine	0.97	0.720	0.914
		Tyrosine	1.06	0.693	0.914
		*N*-acetyltyrosine	1.47	0.051	0.613
		4-hydroxycinnamate	1.83	0.172	0.724
		Tyramine	0.60	0.001	0.075
		m-tyramine	0.99	0.923	0.938
		4-hydroxyphenylpyruvate	2.23	0.012	0.313
		3-(4-hydroxyphenyl)lactate	1.33	0.280	0.762
		Phenol sulfate	1.74	0.016	0.365
		p-cresol sulfate	0.87	0.465	0.874
		o-cresol sulfate	1.41	0.301	0.782
		Dopamine	0.37	0.998	0.947
		Vanillylmandelate (VMA)	1.08	0.392	0.858
		3-methoxytyrosine	1.08	0.967	0.938
		3-methoxytyramine	1.07	0.226	0.748
		3-methoxytyramine sulfate	0.96	0.301	0.782
		3,4-dihydroxyphenylacetate	1.32	0.107	0.724
		Homovanillate (HVA)	1.33	0.040	0.583
		Homovanillate sulfate	0.98	0.565	0.881
		Gentisate	1.37	0.125	0.724
		Phenylpropionylglycine	0.95	0.946	0.938
		3-[3-(sulfooxy)phenyl]propanoic acid	0.66	0.602	0.893
		3-(3-hydroxyphenyl)propionate	1.01	0.903	0.938
		5-hydroxymethyl-2-furoic acid	0.77	0.442	0.861
		2-hydroxyphenylacetate	1.31	0.075	0.721
		Phenylacetylcarnitine	0.82	0.542	0.881
		Dopamine sulfate (1)	0.48	0.963	0.938
		Dopamine sulfate (2)	0.46	0.893	0.938
		p-cresol-glucuronide	0.95	0.704	0.914
		Tyramine *O*-sulfate	0.69	0.202	0.735
		Vanillic alcohol sulfate	0.91	0.271	0.762
		4-hydroxycinnamate sulfate	0.47	0.240	0.748
		3,4-dihydroxyphenylacetate sulfate	0.69	0.548	0.881
	Histidine Metabolism	Histidine	1.10	0.402	0.861
		*N*-acetylhistidine	1.12	0.152	0.724
		1-methylhistidine	1.09	0.712	0.914
		3-methylhistidine	2.02	0.080	0.724
		*N*-acetyl-3-methylhistidine	2.10	0.037	0.576
		*N*-acetyl-1-methylhistidine	1.07	0.482	0.874
		Hydantoin-5-propionic acid	1.01	0.954	0.938
		*trans*-urocanate	1.14	0.280	0.762
		*cis*-urocanate	1.11	0.632	0.905
		Formiminoglutamate	1.03	0.984	0.943
		Imidazole propionate	1.67	0.010	0.290
		Imidazole lactate	1.31	0.280	0.762
		1-methylimidazoleacetate	1.13	0.424	0.861
		4-imidazoleacetate	1.16	0.703	0.914
		*N*-acetylhistamine	0.80	0.554	0.881

## Supplementary Materials

Supplementary materials can be found at www.mdpi.com/1422-0067/18/3/554/s1.

## Abbreviations

hRSV	High-dose resveratrol
RF	Random forest analysis
PEV	Pathway enrichment value
DHA	Docosahexaenoate
ALA	α-linolenic acid
LA	Linoleic acid
MetS	Metabolic syndrome
