# Supplementary material for: Comprehensive Metabolomic Analysis in Blood, Urine, Fat, and Muscle in Men with Metabolic Syndrome: A Randomized, Placebo-Controlled Clinical Trial on the Effects of Resveratrol after Four Months’ Treatment
Source: Int J Mol Sci. 2017 Mar 4;18(3):554. doi: 10.3390/ijms18030554 (PMC5372570; doi:10.3390/ijms18030554)
Supplement: Supplementary file 1 [file ijms-18-00554-s001.zip › ijms-174677 SI.pdf]

Supplementary Appendix

The present appendix describing the Metabolon Platform and Metabolomic Statistical Methods is based on information provided by Metabolon® Inc.:

Metabolon Platform

Sample Accessioning: Following receipt, samples were inventoried and immediately stored at –80 °C. Each sample received was accessioned into the Metabolon LIMS system and was assigned by the LIMS a unique identifier that was associated with the original source identifier only. This identifier was used to track all sample handling, tasks, results, etc. The samples (and all derived aliquots) were tracked by the LIMS system. All portions of any sample were automatically assigned their own unique identifiers by the LIMS when a new task was created; the relationships between these samples were also tracked. All samples were maintained at –80 °C until processed.

Sample Preparation: Samples were prepared using the automated MicroLab STAR® system from Hamilton Company, Reno, NV, USA. A recovery standard was added prior to the first step in the extraction process for QC purposes. To remove protein, dissociated small molecules were bound to proteins or trapped in the precipitated protein matrix, and to recover chemically diverse metabolites, proteins were precipitated with methanol under vigorous shaking for 2 min (Glen Mills GenoGrinder 2000, Clifton, NJ, USA) followed by centrifugation. The resulting extract was divided into five fractions: one for analysis by UPLC-MS/MS with positive ion mode electrospray ionization, one for analysis by UPLC-MS/MS with negative ion mode electrospray ionization, one for LC polar platform, one for analysis by GC-MS, and one sample reserved for backup. Samples were placed briefly on a TurboVap® (Zymark, Hopkinton, MA, USA) to remove the organic solvent. For LC, the samples were stored overnight under nitrogen before preparation for analysis. For GC, each sample was dried under a vacuum overnight before preparation for analysis.

QA/QC: Several types of controls were analyzed in concert with the experimental samples: a pooled matrix sample generated by taking a small volume of each experimental sample (or alternatively, use of a pool of well-characterized human plasma) served as a technical replicate throughout the data set; extracted water samples served as process blanks; and a cocktail of QC standards that were carefully chosen not to interfere with the measurement of endogenous compounds were spiked into every analyzed sample, allowed instrument performance monitoring and aided chromatographic alignment. Tables S8 and S9 describe these QC samples and

standards. Instrument variability was determined by calculating the median relative standard deviation (RSD) for the standards that were added to each sample prior to injection into the mass spectrometers. Overall process variability was determined by calculating the median RSD for all endogenous metabolites (i.e., non-instrument standards) present in 100% of the pooled matrix samples. Experimental samples were randomized across the platform run with QC samples spaced evenly among the injections, as outlined in Figure S1.

Table S8. Description of metabolon QC samples.

Type	Description	Purpose
MTRX	Large pool of human plasma maintained by Metabolon that has been characterized extensively	Ensure that all aspects of the Metabolon process are operating within specifications.
CMTRX	Pool created by taking a small aliquot from every customer sample	Assess the effect of a non-plasma matrix on the Metabolon process and distinguish biological variability from process variability.
PRCS	Aliquot of ultra-pure water	Process blank used to assess the contribution to compound signals from the process.
SOLV	Aliquot of solvents used in extraction	Solvent blank used to segregate contamination sources in the extraction.

Table S9. Metabolon QC standards.

Type	Description	Purpose
RS	Recovery Standard	Assess variability and verify performance of extraction and instrumentation.
DS	Derivatization Standard	Assess variability of derivatization for GC-MS samples.
IS	Internal Standard	Assess variability and performance of instrument.

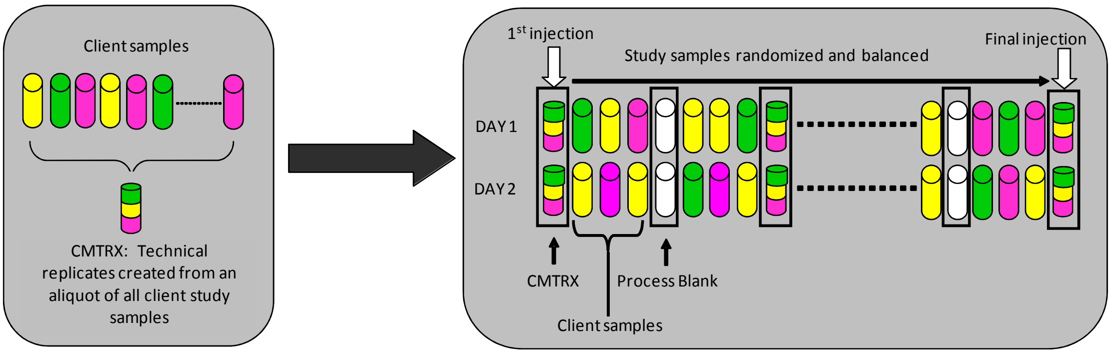

Figure S1. Preparation of client-specific technical replicates. A small aliquot of each client sample (colored cylinders) is pooled to create a CMTRX technical replicate sample (multi-colored cylinder), which is then injected periodically throughout the platform run. Variability among consistently detected biochemicals can be used to calculate an estimate of overall process and platform variability.

Ultrahigh Performance Liquid Chromatography-Tandem Mass Spectroscopy (UPLC-MS/MS): The LC/MS portion of the platform was based on a Waters (Milford, MA, USA) ACQUITY ultra-performance liquid chromatography (UPLC) and a Thermo Scientific (Waltham, MA, USA) Q-Exactive high resolution/accurate mass spectrometer interfaced with a heated electrospray ionization (HESI-II) source and Orbitrap mass analyzer operated at 35,000 mass resolution. The sample extract was dried then reconstituted in acidic or basic LC-compatible solvents, each of which contained eight or more injection standards at fixed concentrations to ensure injection and chromatographic consistency. One aliquot was analyzed using acidic positive ion optimized conditions and the other using basic negative ion optimized conditions in two independent injections using separate dedicated columns (Waters UPLC BEH C18-2.1x100 mm, 1.7 µm). Extracts reconstituted in acidic conditions were gradient eluted from a C18 column using water and methanol containing 0.1% formic acid. The basic extracts were similarly eluted from C18 using methanol and water, however with 6.5 mM Ammonium Bicarbonate. The third aliquot was analyzed via negative ionization following elution from a HILIC column (Waters UPLC BEH Amide 2.1 x 150 mm, 1.7 µm) using a gradient consisting of water and acetonitrile with 10mM ammonium formate. The MS analysis alternated between MS and data-dependent MS2 scans using dynamic exclusion, and the scan range was from 80–1000 m/z. Raw data files are archived and extracted as described below.

Gas Chromatography–Mass Spectroscopy (GC-MS): The samples destined for analysis by GC-MS were dried under vacuum for a minimum of 18 h prior to being derivatized under dried nitrogen using bistrimethyl-silyltrifluoroacetamide. Derivatized samples

were separated on a 5% diphenyl / 95% dimethyl polysiloxane fused silica column (20 m x 0.18 mm ID; 0.18 um film thickness) with helium as carrier gas and a temperature ramp from 60° to 340°C in a 17.5 min period. Samples were analyzed on a Thermo-Finnigan (Waltham, MA, USA) Trace DSQ fast-scanning single-quadrupole mass spectrometer using electron impact ionization (EI) and operated at unit mass resolving power. The scan range was 50–750 m/z. Raw data files are archived and extracted as described below.

Bioinformatics: The informatics system consisted of four major components, the Laboratory Information Management System (LIMS), the data extraction and peak-identification software, data processing tools for QC and compound identification, and a collection of information interpretation and visualization tools for use by data analysts. The hardware and software foundations for these informatics components were the LAN backbone, and a database server running Oracle 10.2.0.1 Enterprise Edition, Redwood Shores, CA, USA.

LIMS: The purpose of the Metabolon LIMS system was to enable fully auditable laboratory automation through a secure, easy to use, and highly specialized system. The scope of the Metabolon LIMS system encompasses sample accessioning, sample preparation and instrumental analysis and reporting, and advanced data analysis. All of the subsequent software systems are grounded in the LIMS data structures. It has been modified to leverage and interface with the in-house information extraction and data visualization systems, as well as third-party instrumentation and data analysis software.

Data Extraction and Compound Identification: Raw data was extracted, peak-identified and QC processed using Metabolon's hardware and software. These systems are built on a web-service platform utilizing Microsoft's .NET technologies, which run on high-performance application servers and fiber-channel storage arrays in clusters to provide active failover and load-balancing. Compounds were identified by comparison with library entries of purified standards or recurrent unknown entities. Metabolon maintains a library based on authenticated standards that contains the retention time/index (RI), mass to charge ratio (*m/z)*, and chromatographic data (including MS/MS spectral data) on all molecules present in the library. Furthermore, biochemical identifications are based on three criteria: retention index within a narrow RI window of the proposed identification, accurate mass match to the library +/- 0.005 amu, and the MS/MS forward and reverse scores between the experimental data and authentic standards. The MS/MS scores are based on a comparison of the ions present in the experimental spectrum to the ions present in the library spectrum. While there may be similarities between these molecules based on one of these factors, the use of all three

data points can be utilized to distinguish and differentiate biochemicals. More than 3300 commercially available purified standard compounds have been acquired and registered into LIMS for distribution to both the LC-MS and GC-MS platforms for determination of their analytical characteristics. Additional mass spectral entries have been created for structurally unnamed biochemicals, which have been identified by virtue of their recurrent nature (both chromatographic and mass spectral). These compounds have the potential to be identified by future acquisition of a matching purified standard or by classical structural analysis.

Curation: A variety of curation procedures were carried out to ensure that a high-quality dataset was made available for statistical analysis and data interpretation. The QC and curation processes were designed to ensure accurate and consistent identification of true chemical entities, and to remove those representing system artifacts, mis-assignments, and background noise. Metabolon data analysts use proprietary visualization and interpretation software to confirm the consistency of peak identification among the various samples. Library matches for each compound were checked for each sample and corrected if necessary.

Metabolite Quantification and Data Normalization: Peaks were quantified using area-under-the-curve. For studies spanning multiple days, a data normalization step was performed to correct the variation resulting from instrument inter-day tuning differences. Essentially, each compound was corrected in run-day blocks by registering the medians to equal one (1.00) and normalizing each data point proportionately (termed the "block correction"; Figure S2). For studies that did not require more than one day of analysis, no normalization is necessary, other than for purposes of data visualization. In certain instances, biochemical data may have been normalized to an additional factor (e.g., cell counts, total protein as determined by Bradford assay, osmolality, etc.) to account for differences in metabolite levels due to differences in the amount of material present in each sample.

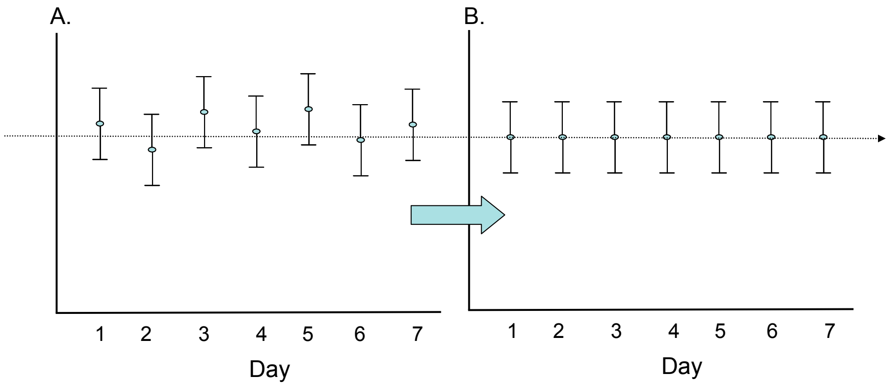

Figure S2. Visualization of data normalization steps for a multiday platform run.

Statistical Methods and Terminology

Statistical Calculations: For many studies, two types of statistical analysis are usually performed: (1) significance tests and (2) classification analysis. Standard statistical analyses are performed in ArrayStudio on log-transformed data. For those analyses not standard in ArrayStudio, the programs R (http://cran.r-project.org/) or JMP are used. Below are examples of frequently employed significance tests and classification methods followed by a discussion of *p*- and *q*-value significance thresholds.

1. **Welch's two-sample *t*-test**

   Welch's two-sample *t*-test is used to test whether two unknown means are different from two independent populations.

   This version of the two-sample *t*-test allows for unequal variances (variance is the square of the standard deviation) and has an *approximate t*-distribution with degrees of freedom estimated using Satterthwaite's approximation. The test statistic is given by $t = (\bar{x}_1 - \bar{x}_2)/\sqrt{s_1^2/n_1 + s_2^2/n_2}$, and the degrees of freedom are given by $\left(\frac{s_1^2}{n_1} + \frac{s_2^2}{n_2}\right)^2 / \left(\frac{\left(\frac{s_1^2}{n_1}\right)^2}{n_1-1} + \frac{\left(\frac{s_2^2}{n_2}\right)^2}{n_2-1}\right)$, where $\bar{x}_1$, $\bar{x}_2$ are the sample means, s_1, s_2, are the sample standard deviations, and n_1, n_2 are the samples sizes from groups 1 and 2, respectively. We typically use a two-sided test (which tests whether the means are different) as opposed to a one-sided test (which tests whether one mean is greater than the other).

2. ***p*-values**

   For statistical significance testing, *p*-values are given. The lower the *p*-value, the more evidence we have that the null hypothesis (typically that two population means are equal) is not true. If "statistical significance" is declared for *p*-values less than 0.05, then 5% of the time we incorrectly conclude the means are different, when actually they are the same.

   The *p*-value is the probability that the test statistic is at least as extreme as observed in this experiment given that the null hypothesis is true. Hence, the more extreme the statistic, the lower the *p*-value and the more evidence the data give against the null hypothesis.

3. ***q*-values**

The level of 0.05 is the false positive rate when there is one test. However, for a large number of tests we need to account for false positives. There are different methods to correct for multiple testing. The oldest methods are family-wise error rate adjustments (Bonferroni, Tukey, etc.), but these tend to be extremely conservative for a very large number of tests. With gene arrays, using the False Discovery Rate (FDR) is more common. The family-wise error rate adjustments give one a high degree of confidence that there are zero false discoveries. However, with FDR methods, one can allow for a small number of false discoveries. The FDR for a given set of compounds can be estimated using the *q*-value (see Storey J and Tibshirani R. (2003) Statistical significance for genome-wide studies. *Proc. Natl. Acad. Sci. USA* 100: 9440-9445; PMID: 12883005).

In order to interpret the q-value, the data must first be sorted by the *p*-value; then we can choose a cutoff for significance (typically $p < 0.05$). The *q*-value gives the false discovery rate for the selected list (i.e., an estimate of the proportion of false discoveries for the list of compounds whose *p*-value is below the cutoff for significance). For Table S10 below, if the whole list is declared significant, then the false discovery rate is approximately 10%. If everything from Compound 079 and above is declared significant, then the false discovery rate is approximately 2.5%.

Table S10. Example of *q*-value interpretation.

Compound	*p*-value	*q*-value
Compound 103	0.0002	0.0122
Compound 212	0.0004	0.0122
Compound 076	0.0004	0.0122
Compound 002	0.0005	0.0122
Compound 168	0.0006	0.0122
Compound 079	0.0016	0.0258
Compound 113	0.0052	0.0631
Compound 050	0.0053	0.0631
Compound 098	0.0061	0.0647
Compound 267	0.0098	0.0939

4. **Random Forest**

Random forest is a supervised classification technique based on an ensemble of decision trees (see Breiman L. (2001) Random Forests. *Machine Learning*. 45: 5-32; http://link.springer.com/article/10.1023%2FA%3A1010933404324). For a given decision tree, a random subset of the data with identifying true class information is selected to build the tree ("bootstrap sample" or "training set"), and then the remaining data, the "out-of-bag" (OOB) variables, are passed down the tree to obtain a class prediction for each sample. This process is repeated thousands of times to produce the forest. The final classification of each sample is determined by computing the class prediction frequency ("votes") for the OOB variables over the whole forest. For example, suppose the random forest consists of 50,000 trees and that 25,000 trees had a prediction for sample 1. Of these 25,000, suppose 15,000 trees classified the sample as belonging to Group A and the remaining 10,000 classified it as belonging to Group B. Then the votes are 0.6 for Group A and 0.4 for Group B, and hence the final classification is Group A. This method is unbiased since the prediction for each sample is based on trees built from a subset of samples that do not include that sample. When the full forest is grown, the class predictions are compared to the true classes, generating the "OOB error rate" as a measure of prediction accuracy. Thus, the prediction accuracy is an unbiased estimate of how well one can predict sample class in a new dataset. Random forest has several advantages: it makes no parametric assumptions, variable selection is not needed, it does not overfit, it is invariant to transformation, and it is fairly easy to implement with R.

To determine which variables (biochemicals) make the largest contribution to the classification, a "variable importance" measure is computed. We use the "Mean Decrease Accuracy" (MDA) as this metric. The MDA is determined by randomly permuting a variable, running the observed values through the trees, and then reassessing the prediction accuracy. If a variable is not important, then this procedure will have little effect on the accuracy of the class prediction (permuting random noise will give random noise). By contrast, if a variable is important to the classification, the prediction accuracy will drop after such a permutation, which we record as the MDA. Thus, the random forest analysis provides an "importance" rank ordering of biochemicals; we typically output the top 30 biochemicals in the list as potentially worthy of further investigation.

5. **Pathway enrichment values**

For each individual pair-wise comparison, Pathway Enrichment displays the number of experimentally regulated compounds relative to all detected

compounds in a pathway, compared to the total number of experimentally regulated compounds relative to all detected compounds in the study. A pathway enrichment value greater than one indicates that the pathway contains more experimentally regulated compounds relative to the study overall, suggesting that the pathway may be a target of interest of the experimental perturbation:

Enrichment: (# of significant metabolites in pathway(k) / total # of detected metabolites in pathway(m)) / (total # of significant metabolites(n) / total # of detected metabolites(N)) (k/m)/(n/N).
